# Equatorial ionization anomaly response to lunar phase and stratospheric sudden warming

**DOI:** 10.1038/s41598-021-94326-x

**Published:** 2021-07-19

**Authors:** Tsung-Yu Wu, Jann-Yenq Liu, Loren C. Chang, Chien‐Hung Lin, Yi-Chung Chiu

**Affiliations:** 1 Center for Astronautical Physics and Engineering, National Central University, Taoyuan, Taiwan; 2 Department of Space Science and Engineering, National Central University, Taoyuan, Taiwan; 3 Center for Space and Remote Sensing Research, National Central University, Taoyuan, Taiwan; 4 Department of Earth Science, National Cheng Kung University, Tainan, Taiwan

**Keywords:** Space physics, Atmospheric science

## Abstract

This study examines the ionosphere response to gravitational forces of the lunar phase and dynamical disturbances of the stratospheric sudden warmings (SSWs). The total electron content (TEC) of global ionosphere maps is employed to examine responses of the equatorial ionization anomaly (EIA) crests to lunar phases and twelve SSW events during 2000–2013. The most prominent feature in the ionosphere is the EIA, characterized by two enhanced TEC crests at low latitudes straddling the magnetic equator, which can be used to observe ionospheric plasma dynamics and structures. Results show that the EIA crest appearance time on new/full moons (first/third quarters) leads (lags) that of the overall 14-year average, which causes a pattern of TEC morning enhancements (suppressions) and afternoon suppressions (enhancements). A statistical analysis shows that SSWs can also significantly cause the early appearance of EIA crests, regardless of the lunar phase. Thus, both lunar phase and SSWs can significantly modulate the appearance time of EIA crest and ionospheric plasma dynamics and structures.

## Introduction

Equatorial ionization anomaly (EIA) is the most pronounced low-latitude ionospheric structure, featured by two dense bands of electron density around ± 12°N magnetic latitude straddling the magnetic equator^1–3^. Stratospheric sudden warmings (SSWs) are remarkable dynamical disturbances in the high-latitude stratosphere during winter^4–6^. During SSWs, the stratospheric polar temperature rapidly increases, the normally eastward winds in the high-latitude stratosphere (60° N and 10 hPa) decelerate, potentially reversing direction, and the structure of the polar vortex changes significantly^4–7^. Recent 10-year studies have shown that lunar tides get enhanced as a result of SSWs related changes in the background lower and middle atmosphere, which then goes on to impact the ionosphere through the dynamo mechanism^7–18^. Goncharenko et al.^14^ studied the ionosphere response to SSWs and observed the pattern of total electron content (TEC) enhancements in the morning sector and suppressions in the afternoon sector at EIA latitudes during SSW occurred on 19–31 January 2009.

Meanwhile, scientists^19, 20^ find that the lunar phase, which is defined as the relative position among the Sun, the Moon, and the Earth, can significantly modify the EIA appearance. Wu et al.^19^ examine the ionospheric EIA crests response to the lunar phase during the 18-year period of 2000–2017 using the global ionosphere map (GIM) of TEC. They find that the EIA crests lead those of the overall 18-year average by about 20–40 min on new/full moon, which result in a prominent EIA with TEC increasing during 08:00–15:00 SLT (solar local time) and decreasing during 1600–22:00 SLT.

Since both lunar phases and SSWs can result in similar morning enhancement and afternoon suppression features, this study utilizes GIM TECs to study the ionospheric EIA TEC response to the lunar phases and SSWs. Based on CP07^4^, Butler et al.^21^ report 12 SSWs during the 14-year period of 2000–2013, while lunar calendar shows that there are 112 semimonthly lunar periods (14.76 days) in November–March of the same 14-year period. Here we show that response of EIA crests to the lunar phase from above is stronger than that to the SSW from below.

## Results

### Stratospheric sudden warming effects on the EIA

**Figure 1:**
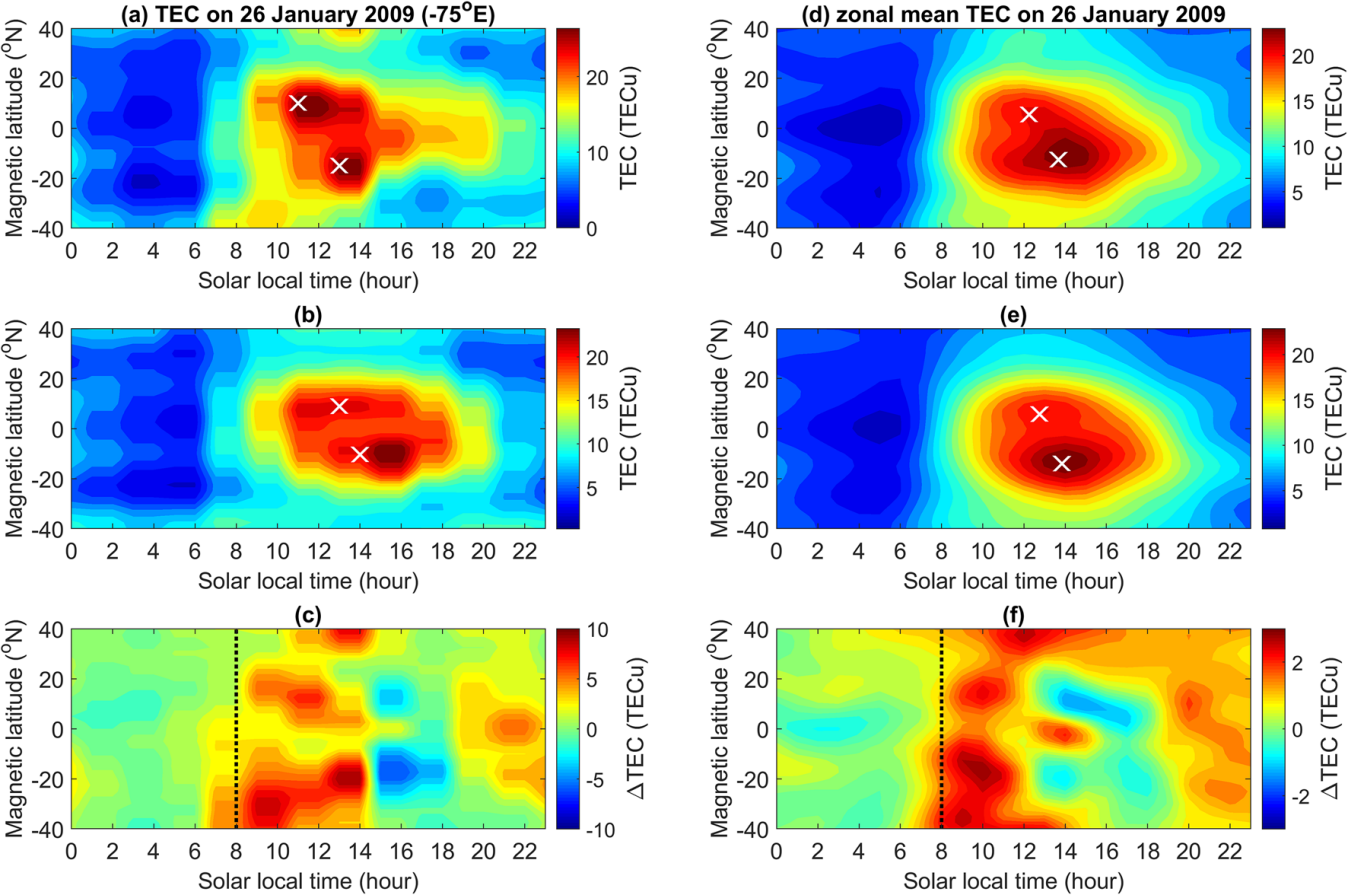
LTT plots of the GIM TEC and their associated differences along − 75° E longitude and the zonal mean on 26 January 2009. LTT along − 75° E longitude on (**a**) 26 January 2009, (**b**) associated median of 7 days before and after, and (**c**) their difference, respectively. (**d–f**) zonal mean LTT of 24 longitudes of the globe on 26 January 2009, reference, and their difference, respectively. White cross symbols denote the corresponding EIA crests in Northern and Southern Hemispheres. Dashed black lines denote 08:00 SLT. Note that the EIA crest of the reference is computed by averaging over the individual crest 7 days before and after 26 January 2009. The TEC unit (TECu) is 10^16^ #/m^2^.

Figure 1a–c display the latitude-time-TEC (LTT) plots along − 75° E (i.e., 75°W) longitude on the SSW day of 26 January 2009 (a new moon day of the lunar phase) (TEC_SSW_), associated reference (TEC_ref_), and difference (ΔTEC = TEC_SSW_ − TEC_ref_). TEC_ref_ is calculated by a moving median at each SLT 7 days before and after a certain observation day (here, the SSW day), the difference between observation and reference is further computed (i.e. ΔTEC). In Fig. 1, the northern and southern EIA crests appear at 11:00 SLT (with the strength of 27.7 TECu (1 TECu = 10^16^ #/m^2^)) and 13:00 SLT (27.2 TECu) on the SSW day (Fig. 1a), as well as 13:00 SLT (26.5 TECu) and 14:00 SLT (26.1 TECu) on the reference day (Fig. 1b), respectively. Figure 1c shows that the TECs are enhanced in the morning and suppressed in the afternoon, which suggests that the EIA crests appear earlier on the SSW day. The TEC enhancement/suppression pattern is consistent with previous findings on SSW effects^14^. To observe global GIM TEC responses to SSWs, we compute the zonal mean of 24 longitudes around the globe with an interval of 15° (i.e. 1 h) and plot the averaged LTT on the SSW day, reference, and their difference. The northern and southern EIA crests appear at 12:15 SLT (22.7 TECu) and 13:39 SLT (22.8 TECu) on the SSW day (Fig. 1d), as well as at 12:45 SLT (21.1 TECu) and 13:50 SLT (22.3 TECu) on the reference day (Fig. 1e), respectively. Figure 1a–b and d–e confirm that the EIA crests appear earlier on the SSW day, while Fig. 1c,f demonstrate that the morning enhancements and afternoon suppressions on the SSW day are global effects.

### Lunar phase effects on the EIA

**Figure 2:**
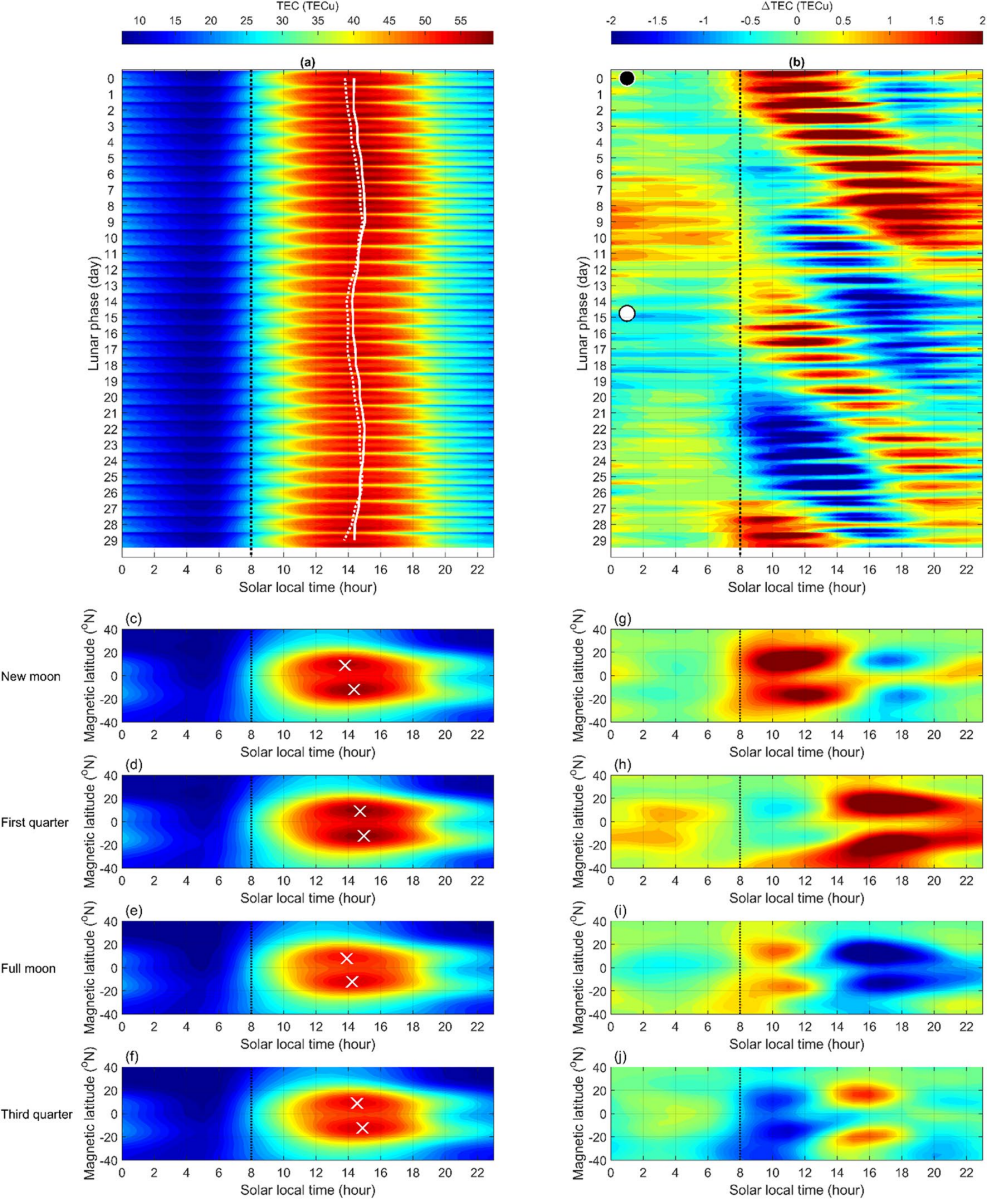
LTT plots of TEC and associated differences versus lunar phase during 2000–2013. (**a**) Averaged LTT plots in various lunar phases and (**b**) their departures from the overall means within ± 20° magnetic latitude during November–March in 2000–2013. (**c**–**f**) Averaged LTT plots on new moon (day 0), first quarter (day 7), full moon (day 14), and third quarter (day 22) (**g**–**j**) as well as their departures accordingly. Solid and dotted white curves denote the EIA crest appearance time in Northern and Southern Hemispheres, respectively. White cross symbols denote the corresponding EIA crests in Northern and Southern Hemispheres. Dashed black lines denote 08:00 SLT.

We further study the response of the zonal mean TEC and EIA crests within ± 20° magnetic latitude to the lunar phase during November–March in the period of 2000–2013. The Lunar Calendar (https://www.timeanddate.com/) is used to find the lunar phase day in the studied period. Figures 2a and 2b illustrate LTTs in various lunar phases and their departures from the averaged of the overall means (i.e. ΔTEC), respectively. LTTs in various lunar phases are similar to each other, and however the EIA crests versus lunar phase reveals a clear sinusoidal variation, which indicates early EIA appearances on the new and full moon but late ones on first and third quarter (Fig. 2a). Figure 2c–f further depict the EIA crests of the new moon, first quarter, full moon, and third quarter appear around 13:48 (14:21), 14:43 (14:58), 13:55 (14:15), and 14:33 (14:54) SLT in the Northern (Southern) Hemisphere, respectively. These results show that the semimonthly lunar periodicity can respectively advance and delay the appearance of EIA crests during the new/full moon and first/third quarter. Meanwhile, Figs. 2g–j depict that ΔTEC prominently increases (decreases) at about 08:00–15:00 SLT and decreases (increases) at around 1600–22:00 SLT during new/full moon (first/third quarter). Thus, the TEC is enhanced (suppressed) in the morning and suppressed (enhanced) in the afternoon around new/full moon (first/third quarter) days (Fig. 2b,j–j). It is clear that in comparing the reference, the TEC starts increasing (decreasing) at about 08:00 SLT within ± 2 days of the new or full moon (first or third quarter). Note that the SSW day in Fig. 1 is right on a new moon day, and its EIA crest behavior is very similar to those around the new moon shown in Fig. 2. Therefore, it is interesting to find that the TEC enhancement in the morning and suppression in the afternoon (i.e. the EIA earlier appearance) results from lunar phase and/or SSW.

### Lunar phase versus stratospheric sudden warming

**Figure 3:**
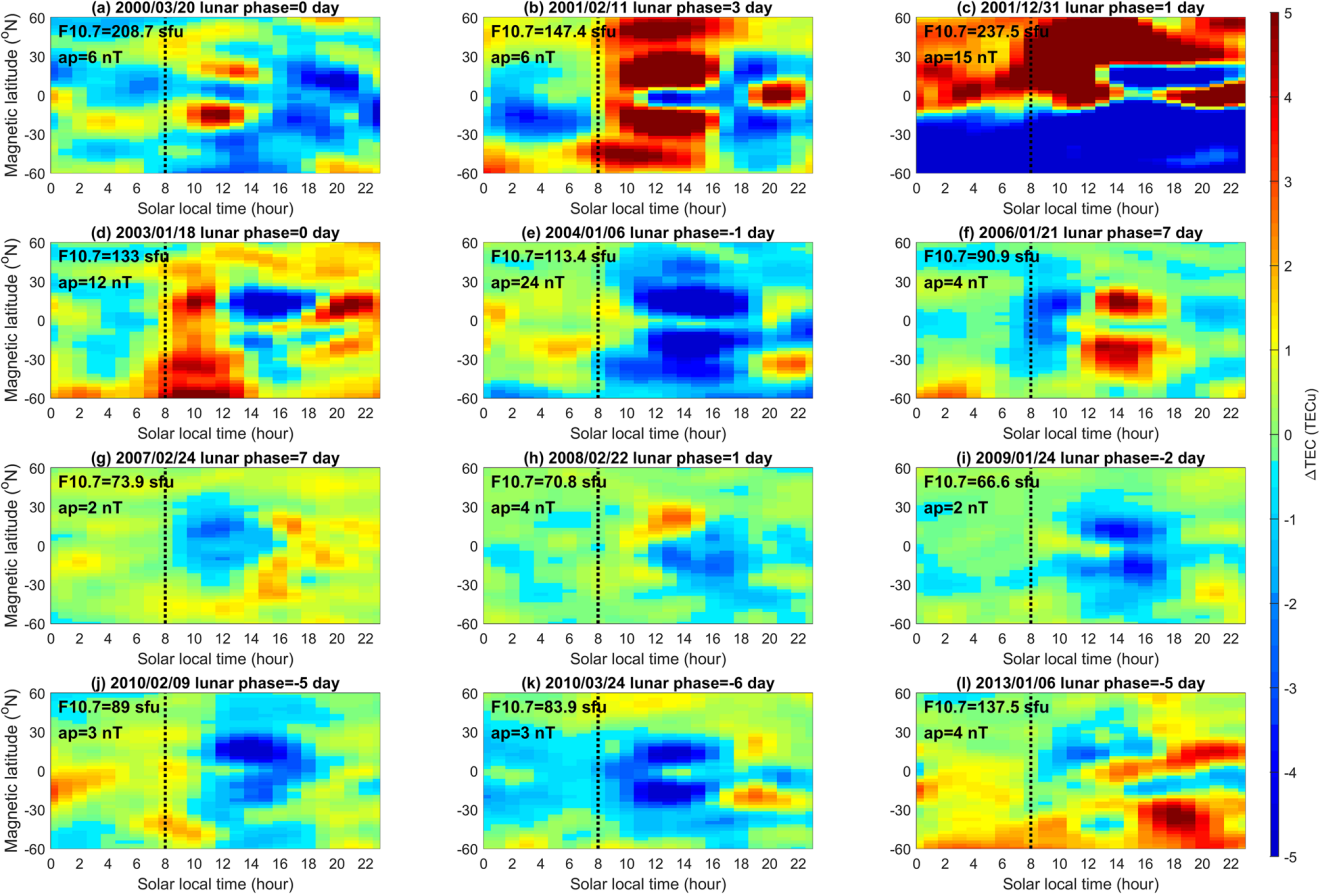
LTT ΔTEC plots of the 12 SSW events of CP07. Dashed black lines denote 08:00 SLT. Daily solar flux and ap index for each SSW event are denoted accordingly.

**Figure 4:**
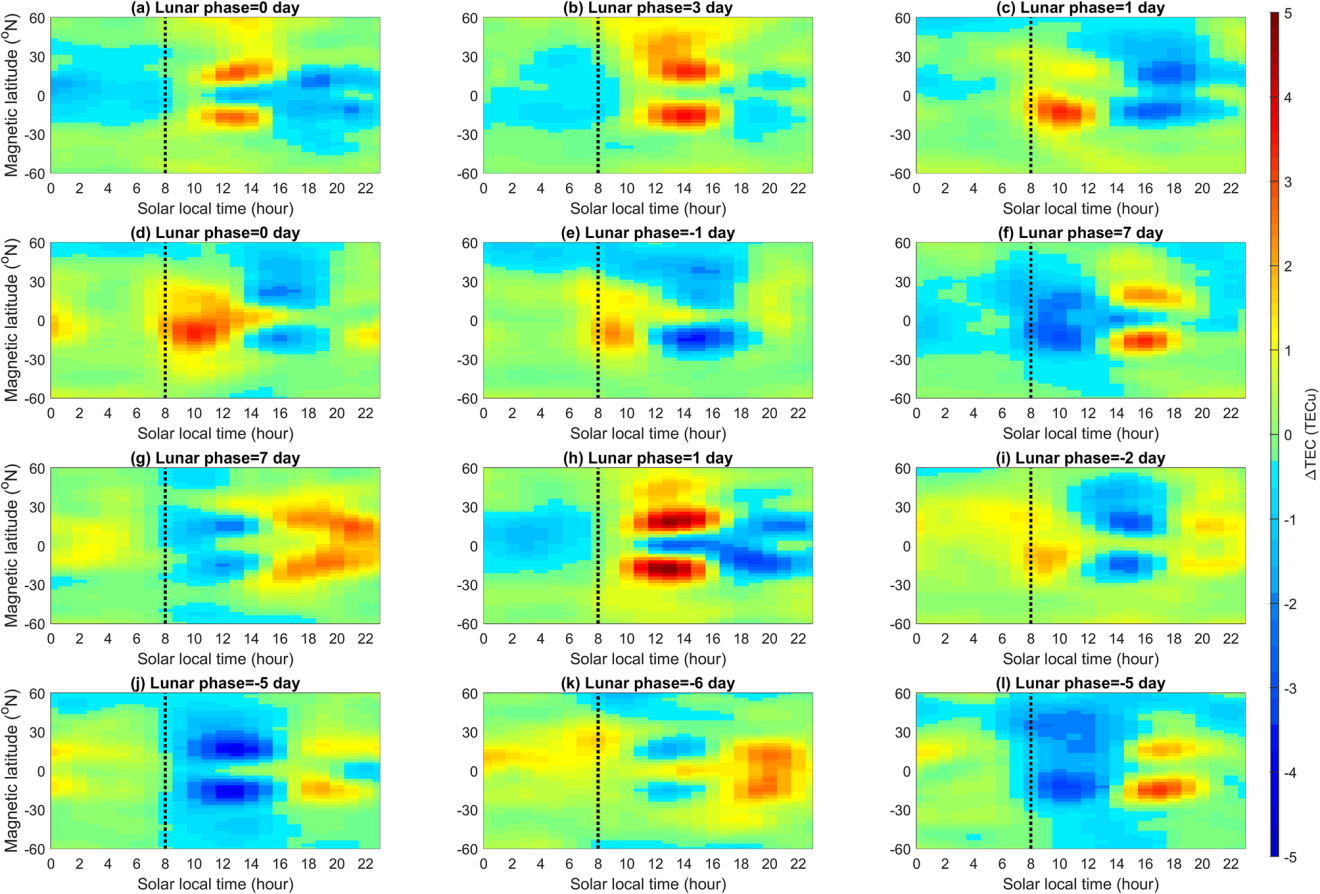
LTT plots on the same lunar phase day as the 12 CP07 SSW events but in the other 13 years. Each plot has been averaged over the 13 years.

To compare the response of EIA crests to SSWs and that to the lunar phase in detail, we examine the 12 SSW events and corresponding lunar phases during 2000–2013 reported in Butler et al.^21^. The 12 SSWs occur in various lunar phases, which allow us to find whether SSWs result in the morning enhancement and the afternoon suppression. Again, the Lunar Calendar (https://www.timeanddate.com/) is used to find the lunar phase day of the 12 SSWs. Since lunar gravitational effects on new moon and full moon are almost the same, lunar phase day 0 stands for the new or full moon day, where the minus “−” and the plus “+” sign mean the event occurs before and after that day, respectively. Figure 3 shows that for the SSWs occurring around new/full moon, Events A (lunar phase day 0), B (3), C (1), D (0), E (− 1), H (1), and I (− 2), the EIA TEC indeed tends to be enhanced in the morning and suppressed in the afternoon sector. By contrast, for those occurring around first/third quarter, Events F (lunar phase day 7), G (7), J (− 5), K (− 6), and L (− 5) show suppressions in the morning and increases in the afternoon sector. We further examine the TEC of EIA crests on the corresponding lunar phase day of each SSW event for the other 13 years (i.e. excluding the day of the SSW year). Figure 4 displays the corresponding 13-year averaged LTT plots of each event (Supplementary material), which shows that the pattern in each panel of Fig. 3 and that in Fig. 4 generally are very similar. The similarities among Figs. 2, 3, and 4 strongly suggest the lunar phase or semimonthly lunar periodicity to the EIA crest appearance time, which results in TEC changes in the morning and the afternoon, playing an important role.

**Figure 5:**
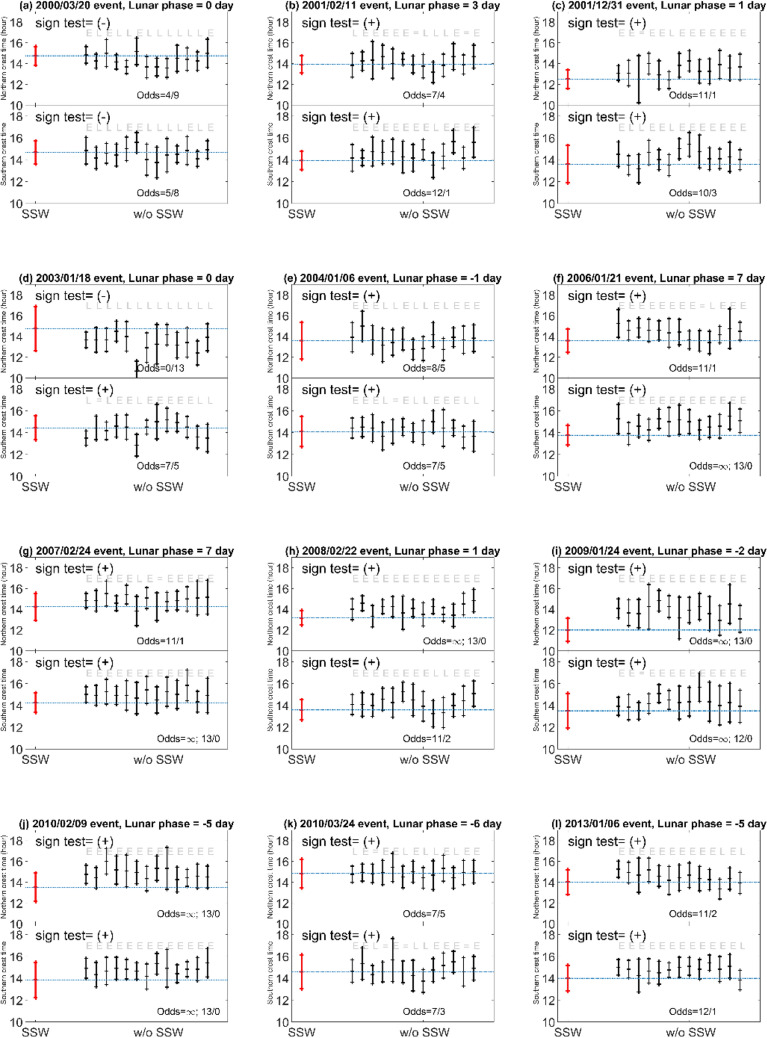
The EIA crest appearance times on day with and without SSW events. The EIA crest appearance times of the 24 longitudes on the SSW event day (red segment) and associated references lunar day without (w/o) SSW in the other 13 years (black segments) in the both hemispheres. Each segment consists of 24 data points in the 24 longitudes of the globe. Two end ticks of the segment stand for one standard deviation, while the center tick denotes the mean. E and L indicate the mean of the EIA crest appearance time of the event being earlier and later than the associated reference mean, respectively. If it's a tie, a sign of “ = ” is denoted. When tie, it will not be included the ratio calculation.

To see if an SSW could cause the early appearance of EIA crests, we compare EIA crest appearance times along the 24 longitudes across the entire 14 years in both hemispheres. We first isolate the same lunar day as an SSW event for the other 13 years without (w/o) SSWs, which are considered as the associated references, and compute the mean of EIA crest appearance times of the 24 longitudes in both hemispheres for each event and its 13 references. Figure 5 shows the mean of the EIA crest appearance times of each event and that of the associated 13 references. We further examine the mean EIA crest appearance time of the 24 longitudes of each SSW event and compare to that of the 13 reference year one-by-one. If the mean time of SSW event year is earlier (later) than the reference year, it is denoted as E (L). Odds in the ratio of numbers of E to those of L are then calculated. If the odds are greater than 1, the SSW event can cause early EIA crest appearance, regardless the lunar phase of the event. Finally, we examine the odds of the 12 SSW events being greater than 1. In the Northern Hemisphere, the odds of Events H, I, and J are infinite (∞; 13/0), while those of Events B, C, E, F, G, K and L are generally much greater than 1. Therefore, the sign test of the 12 SSW odds being earlier to later is 5 (= 10/2). The events with the odds greater than 1 in the Southern Hemisphere generally are the same as those in the Northern Hemisphere, except Event D, which is also greater than 1. Thus, the sign test is 11 (= 11/1) in the Southern Hemisphere. If we remove Event C, due to that event being affected by a magnetic storm of Dst < − 58 nT, the sign test becomes 4.5 (= 9/2) in Northern and 10 (= 10/1) in the Southern Hemisphere. Since these sign test among the 12 SSW odds are much greater than the rejection value^22^, which is 2.5 under a significance level of 0.01, SSWs can prominently result in early appearances of EIA crests. We further find the average of time differences resulting from the SSWs by subtracting the mean of the EIA crest appearance time of each event from that of associated 13 references for the overall 12 events, which illustrates that SSWs could advance the EIA crest appearance time by about 0.47 h (Table 1). Detailed information of the above results is summarized in Table 1, which lists the event date, the lunar phase day, the EIA crest appearance time with (w)—without (w/o) SSW day, and the ratio of the EIA crest on the SSW day being earlier to later (E/L) than that of the reference.

**Table 1:** SSW Events during 2000–2013.

Event	yyyy/mm/dd	Lunar Phase (day)	EIA Crest Time w-w/o SSW (SLT)	Ratio of years being Earlier to Later (E/L)	
A	2000/03/20	0	14.75–14.32	4/9	Northern
			14.67–14.54	5/8	Southern
B	2001/02/11	3	13.92–14.10	**7/4**	
			13.92–14.54	**12/1**	
C	2001/12/31	1	12.50–13.37	**11/1**	
			13.58–14.22	**10/3**	
D	2003/01/18	0	14.75–13.34	0/13	
			14.42–14.24	**7/5**	
E	2004/01/06	-1	13.58–13.60	**8/5**	
			14.08–14.21	**7/5**	
F	2006/01/21	7	13.58–14.36	**11/1**	
			13.75–14.79	**∞**; **13/0**	
G	2007/02/24	7	14.25–14.86	**11/1**	
			14.25–14.99	**∞; 13/0**	
H	2008/02/22	1	13.17–14.01	**∞; 13/0**	
			13.58–14.21	**11/2**	
I	2009/01/24	-2	12.00–13.85	**∞; 13/0**	
			13.50–14.29	**∞; 12/0**	
J	2010/02/09	-5	13.50–14.85	**∞; 13/0**	
			13.83–14.79	**∞; 13/0**	
K	2010/03/24	-6	14.83–14.85	**7/5**	
			14.58–14.78	**7/3**	
L	2013/01/06	-5	14.00–14.46	**11/2**	
			14.00–14.78	**12/1**	

Bold character stands for that the ratio of years being earlier to later (E/L) is greater than 1.

Day 0 stands for the new or full moon day, where a minus “−” sign means the event occurs before that day.

The EIA Crest Time of w/o SSW is averaged over the corresponding days of the other 13 w/o SSW years (i.e., total w/o SSW reference).

## Discussion

Goncharenko et al.^14^ studied the ionosphere response to SSWs and observed the pattern of TEC enhancements in the morning sector and suppressions in the afternoon sector at EIA latitudes along − 75° E longitude during 19–31 January 2009 (i.e. around Event I; lunar phase day − 2) and 21–30 January 2008 (i.e. around Event H; lunar phase day 1). The above pattern of morning enhancements and afternoon suppressions along − 75° E longitude in GPS TEC on 26 January 2009 reported by Goncharenko et al. ^14^ agree well with that in GIM TEC (Fig. 1). Figure 1 further demonstrates that the 24 longitudes can be employed to examine ionospheric SSW signatures across the globe. The sinusoidal variation in Fig. 2 reveals that the EIA crests on new/full moon (first/third quarter) lead (lag) those of the overall 14-year average, which consequently results in morning enhancements and afternoon suppressions. Note that the sequential patterns of ΔTEC enhancements and suppressions during 19–31 January 2009 and 21–30 January 2008 shown in the previous study^14^ agree well with the patterns of about the new moon and full moon in Fig. 2; of Event I (Fig. 3i) and Event H (Fig. 3h); and of Fig. 4i,h without SSWs. Note that the pattern of Event F occurring on lunar phase day 7 shown in Fig. 3f also agrees well with that of the first and third quarter shown in Fig. 2. These agreements confirm that the semimonthly lunar periodicity plays an important role.

The statistical analyses of odds and sign tests in Fig. 5 and Table 1 show that SSWs can significantly cause early appearances of EIA crests. The agreement between the previous^14^ and current studies^7, 11, 14–18^ indicates that owing to SSWs, the interactions between planetary waves and tides could alter the tides from the middle to the upper atmosphere, and thus significantly modify the ionospheric EIA. Figure 2 shows that the lunar phase results in the EIA crest appearance time during new/full moons leading that of first/third quarters by about 1 h, while Fig. 5 and Table 1 show that the SSWs cause the EIA crests advancing by about 0.47 h. The patterns in Fig. 3 are very similar to those in Fig. 4 accordingly. These results strongly suggest that the lunar phase can significantly modulate the EIA crests and dominate the pattern of TEC enhancements/suppressions, while SSWs can prominently advance EIA crest appearance time regardless lunar phase. In conclusion, the lunar phase from above can predominantly advance or delay the EIA crest appearance times and TEC enhancement/suppression patterns, while SSWs from below could significantly result in early appearances of the ionospheric EIA crests.

## Methods

Data used in this study are the total electron content (TEC), date of the lunar phase, and date of stratospheric sudden warming (SSW). The TEC is defined as the total number of electrons integrated between ground-based receiver and GNSS (global navigation satellite system) satellites, along a tube of one meter squared cross section. Here, the global ionosphere map (GIM) of TEC is used to study the ionospheric equatorial ionization anomaly (EIA) response to the lunar phases and 12 SSWs during 14-year period of 2000–2013. The GIM TEC has been routinely published by the Center for Orbit Determination in Europe (CODE, ftp://ftp.aiub.unibe.ch/) with temporal resolution of 2 h and spatial resolution of 2.5° in latitude by 5.0° in longitude. The Lunar Calendar (https://www.timeanddate.com/) is used to find the lunar phase day during the 14-year period of 2000–2013. The 12 SSW events during 2000–2013 were reported in Butler et al.^21^. The daily solar flux and ap index for each SSW event are published by Space Physics Data Facility of NASA (National Aeronautics and Space Administration, https://omniweb.gsfc.nasa.gov/html/ow_data.html).

### Latitude-time-TEC plot along a certain longitude

To observe the TEC response to the lunar phases and SSWs along a certain longitude, the plot of latitude-time-TEC (LTT) at various SLTs (solar local times) along the longitude is derived. Figure 1a–c display the LTT plots along − 75° E longitude on the SSW day of 26 January 2009 (a new moon day of the lunar phase) (TEC_SSW_), associated reference (TEC_ref_), and difference (ΔTEC = TEC_SSW_ − TEC_ref_). TEC_ref_ is calculated by a moving median at each SLT 7 days before and after a certain SSW observation day, the difference between observation and reference is further computed (i.e. ΔTEC). The reason why TEC_ref_ is calculated by a moving median at each SLT 7 days before and after a certain observation day is because of close to the semimonthly lunar period of 14.76 days, which allows us to extract the lunar phase signature. By using the same approach, Liu et al.^20^ study the lunar phase signatures on ionospheric solar eclipse signatures on 21 August 2017. They show that the lunar tide signature can be enhanced when the reference is constructed by using ± 7 days (semimonthly lunar period) or longer windows. Once again, the above processes have been conducted under the same solar local time but over a full semimonthly period, thus, which shall lead effects/signatures of the solar signature being removed and those of the lunar phase signature being enhanced.

### Latitude-time-TEC plot of the globe

To observe the global TEC response to the lunar phases and SSWs, the LTT at each fixed SLT is averaged over 24 longitudes of the globe with an interval of 15°. Likewise, the associated reference and difference are constructed accordingly. Figure 1d–f display the LTT plots of the globe on the SSW day of 26 January 2009 (TEC_SSW_), associated reference (TEC_ref_), and difference (ΔTEC).

### TEC response to lunar phase

By averaging TEC and ΔTEC on each lunar phase during the entire 14 years, we can examine the coherent of TEC to the lunar phase in detail. In addition to the TEC, the appearance time of the equatorial ionization anomaly (EIA) crest is utilized for statistical analysis. EIA crests are extracted from each LTT by determining the maximum TEC during 08:00 SLT-20:00 SLT. To discriminate the SSW effects from influences of the lunar phase, the EIA crest time on the same lunar phase day with and without SSW are compared one-by-one (Fig. 5).

### TEC response to SSW

Similar to Fig. 1f, ΔTEC of the globe with and without the SSW events are plotted in Figs. 3 and 4, respectively.

### Corresponding reference of a SSW event

The corresponding lunar phase for the other 13 years excluding that day of the SSW year based on both solar and lunar calendar. First, find the same solar date of each SSW events in the other 13 years. Second, shift the date backward or forward to match the lunar phase to have the same lunar phase of the SSW event. Taking Event B (2001/02/11) as example, the SSW day is on 11 February 2001 and its lunar phase is 3. Based on the lunar calendar, we however find that the lunar phase day of 11 February 2002 is − 1. We then shift it by 4 days forward (15 February 2002) to have the same lunar phase of Event B (lunar phase day 3). Therefore, we yield a corresponding reference with a similar season and the same lunar phase of Event B in 2002. We repeat similar process for the entire 13 years to construct the reference without SSW for Event B.

### Separation of SSW and lunar phase effects on the EIA

Figure 5 illustrates the EIA crest appearance times on the same lunar phase day with and without SSW for each of the 12 events. Owing to the same lunar phase day, the pure SSW effects can be obtained by subtracting the mean EIA appearance time with SSW event by that without (the reference) (Table 1). Results show that the separated or pure SSW effect can further advance EIA appearance time by about 0.47 (= (0.43 + 0.52)/2) hours. (Supplementary material).

### Odds

Odds provide a measure of the likelihood of a particular outcome. They are calculated as the ratio of the number of events that produce the outcome to the number that don't. For each SSW event, we first identify the averaged EIA crest time over the 24 longitudes in both hemispheres on the SSW day and that on the same lunar phase day in the rest other 13 years (Table S1). We then calculate odds as the ratio of the EIA crest time of the SSW day earlier (E) to later (L) than that of the rest 13 years. The ratio of E/L (odds) greater (less) than 1 is defined as “plus” (“minus”), which indicates that EIA crest time is early (later) activated by the SSW event (Table 1). We further find the sign test (or odds) for the entire 12 SSW events. If number of the pluses is greater than that of minuses, it can be claimed that SSWs can significantly advance the EIA crest time.

## Supplementary Information

Supplementary Information.

## Data Availability

The total electron content (TEC) of global ionosphere maps (GIMs) is provided by the Center for Orbit Determination in Europe (CODE) (ftp://ftp.aiub.unibe.ch/). The lunar phase is obtained from Lunar Calendar (https://www.timeanddate.com/). The daily solar flux and ap index are published by Space Physics Data Facility of NASA (National Aeronautics and Space Administration, https://omniweb.gsfc.nasa.gov/html/ow_data.html).

## References

[1] Appleton EV (1946). Two anomalies in the ionosphere. Nature.

[2] Rishbeth H (2000). The equatorial F-layer: Progress and puzzles. Ann. Geophys..

[3] Lin CH, Liu JY, Fang TW, Chang PY, Tsai HF, Chen CH, Hsiao CC (2007). Motions of the equatorial ionization anomaly crests imaged by FORMOSAT-3/ COSMIC. Geophys. Res. Lett..

[4] Charlton AJ, Polvani LM (2007). A new look at stratospheric sudden warmings. Part I: climatology and modeling benchmarks. J. Clim..

[5] Schoeberl MR (1978). Stratospheric warmings: observations and theory. Rev. Geophys..

[6] Limpasuvan V, Thompson DW, Hartmann DL (2004). The life cycle of the Northern Hemisphere sudden stratospheric warmings. J. Clim..

[7] Zhang X, Forbes JM (2014). Lunar tide in the thermosphere and weakening of the northern polar vortex. Geophys. Res. Lett..

[8] Fejer BG (2010). Lunar-dependent equatorial ionospheric electrodynamic effects during sudden stratospheric warmings. J. Geophys. Res..

[9] Forbes JM, Zhang X (2012). Lunar tide amplification during the January 2009 stratosphere warming event: observations and theory. J. Geophys. Res..

[10] Yamazaki Y, Richmond AD, Yumoto K (2012). Stratospheric warmings and the geomagnetic lunar tide: 1958–2007. J. Geophys. Res..

[11] Yamazaki Y (2013). Large lunar tidal effects in the equatorial electrojet during northern winter and its relation to stratospheric sudden warming events. J. Geophys. Res. Space Phys..

[12] Pedatella NM, Maute A (2015). Impact of the semidiurnal lunar tide on the midlatitude thermospheric wind and ionosphere during sudden stratosphere warmings. J. Geophys. Res. Space Phys..

[13] Siddiqui TA, Maute A, Pedatella N, Yamazaki Y, Lühr H, Stolle C (2018). On the variability of the semidiurnal solar and lunar tides of the equatorial electrojet during sudden stratospheric warmings. Ann. Geophys..

[14] Goncharenko LP, Coster AJ, Chau JL, Valladares CE (2010). Impact of sudden stratospheric warmings on equatorial ionization anomaly. J. Geophys. Res..

[15] Yue X (2010). Global ionospheric response observed by COSMIC satellites during the January 2009 stratospheric sudden warming event. J. Geophys. Res..

[16] Lin CH (2012). Observations of global ionospheric responsesto the 2009 stratospheric sudden warming event by FORMOSAT-3/COSMIC. J. Geophys. Res..

[17] Jin H (2012). Response of migrating tides to the stratospheric sudden warming in 2009 and their effects on the ionosphere studied by a whole atmosphere-ionosphere model GAIA with COSMIC and TIMED/SABER observations. J. Geophys. Res..

[18] Lin JT (2019). Revisiting the modulations of ionospheric solar and lunar migrating tides during the 2009 stratospheric sudden warming by using global ionosphere specification. Space Weather.

[19] Wu TY, Liu JY, Lin CY, Chang LC (2020). Response of ionospheric equatorial ionization crests to lunar phase. Geophys. Res. Lett..

[20] Liu JY, Wu T-Y, Sun YY, Pedatella NM, Lin CY, Chang LC (2020). Lunar tide effects on ionospheric solar eclipse signatures: the August 21, 2017 event as an example. J. Geophys. Res. Space Phys..

[21] Butler AH (2015). Defining sudden stratospheric warmings. Bull. Am. Meteor. Soc..

[22] Conover JW (1999). Practical Nonparametric Statistics.

