# Supplementary material for: Equatorial ionization anomaly response to lunar phase and stratospheric sudden warming
Source: Sci Rep. 2021 Jul 19;11:14695. doi: 10.1038/s41598-021-94326-x (PMC8289839; doi:10.1038/s41598-021-94326-x)
Supplement: Supplementary file 1 — Supplementary Information. [file 41598_2021_94326_MOESM1_ESM.docx]

**Supplementary Material for “Equatorial ionization anomaly response to lunar phase and stratospheric sudden warming”**

**Tsung-Yu Wu^1,2^, Jann-Yenq Liu^1,2,3^*, Loren C. Chang^1,2^, Chien-Hung Lin^4^, and Yi-Chung Chiu^2^**

^1^Center for Astronautical Physics and Engineering, National Central University, Taiwan

^2^Department of Space Science and Engineering, National Central University, Taiwan

^3^Center for Space and Remote Sensing Research, National Central University, Taiwan

^4^Department of Earth Science, National Cheng Kung University, Tainan, Taiwan

*****email: jyliu@jupiter.ss.ncu.edu.tw

**Note on Supplemental Material**

The following sections are included:

1. Latitude-Time total electron content: Figs. S1 and S2
2. Lunar phase signature: Figs. S3 and S4
3. Equatorial ionization anomaly crest: Fig. S5
4. Odds.
5. Appearance times of EIA crests versus various lunar phases: Fig. S6
6. LTT plots of each comparison years: Fig. S7
7. Comparison of EIA crest time between with and without SSW: Fig. S8
8. SSW Events and Their Reference Dates: Table S1

To clarify the data process of total electron content to the lunar phase and stratospheric sudden warming, the method is discussed (Figs. S1-S5). To see if an SSW could result in the early appearance of EIA crests under the same lunar phases, the corresponding days, which are on the same lunar day with the nearest solar day in the other 13 years, are isolated (Table S1), which displays the 12 SSW events and their reference days.

1. **Latitude-Time total electron content**

To observe lunar phase signature of TEC, a 14-day moving median is computed. Figure 1b displays the reference for the TEC along the longitude of -75°E on 26 January 2009, which is the median of 7 days before and after 26 January 2009 (Fig. S1). Since lunar phase and SSW are global effect, the average of LTTs in TEC_ssw_ and TEC_ref_ over 24 longitudes are conducted. Figure S2 displays all of the 24 LTTs on 26 January 2009, which will be averaged TEC (i.e. Fig. 1d). Similar, Figure 1f is consisted of 24 LTTs on SSW day and associated 312 (=24x13) LTTs on reference days.


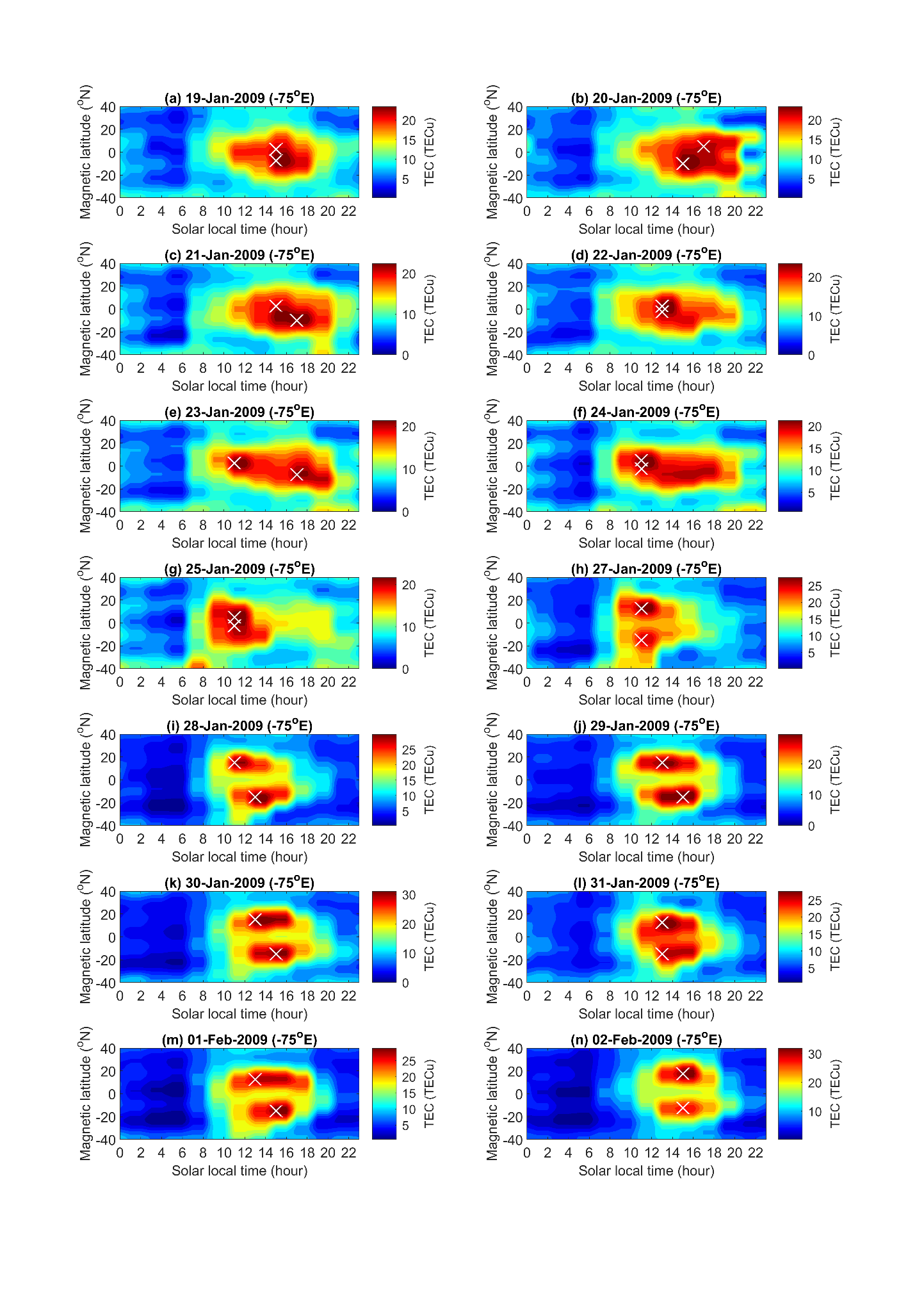


**Fig. S1** 14 LTTs along 75°E for calculating the reference of LTT on 26 Jan 2009.


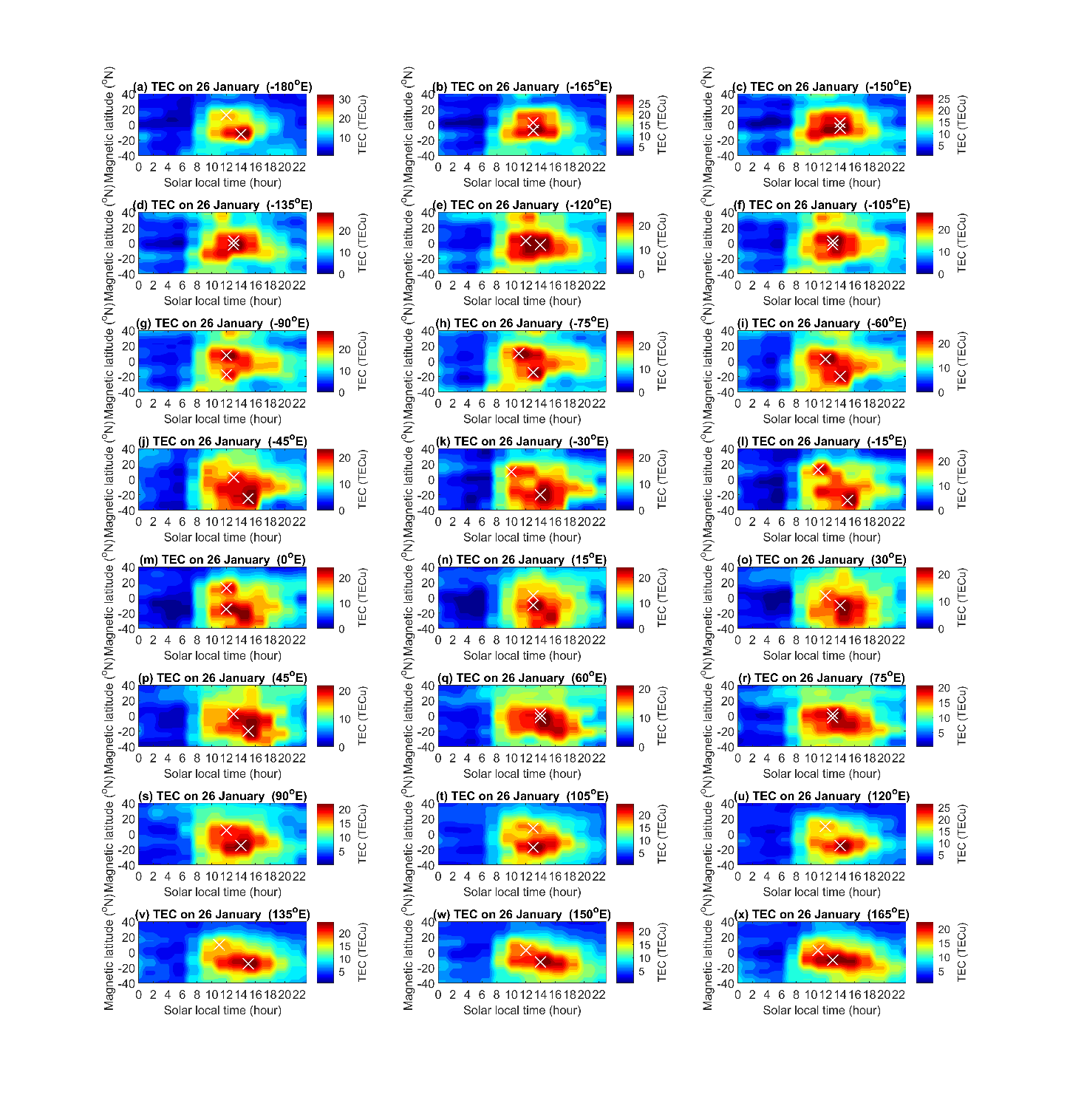


**Fig. S2** 24 LTTs cross the globe for calculating zonal mean TEC.

1. **Lunar phase signature**

In this study, there are 1966-days GIM TEC and 66 new moon days during November-March in the period of 2000–2013. Figure S3 displays the lunar phase day of each studied year, and 0 stand for new moon or full moon day. To find the coherent lunar phase signature, daily averaged TEC and ΔTEC on each lunar phase during the 14 years is computed (Fig. 2). The Lunar calendar is also used to isolate SSW effect by comparing GIM TEC with and without SSW on the same lunar phase day (Figs. 3 and 4).


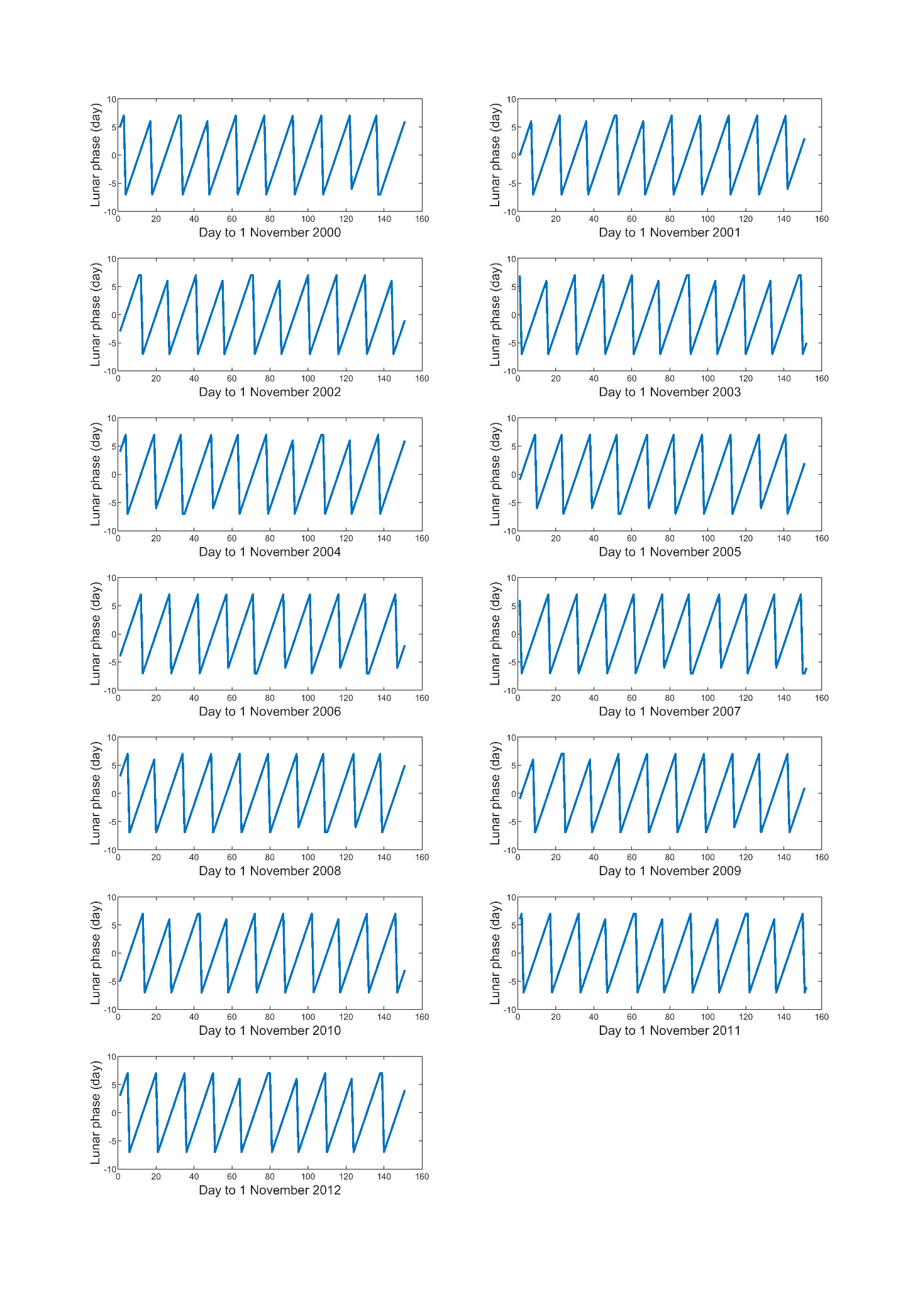


**Fig. S3** Lunar phase from November to March during 2000–2012.


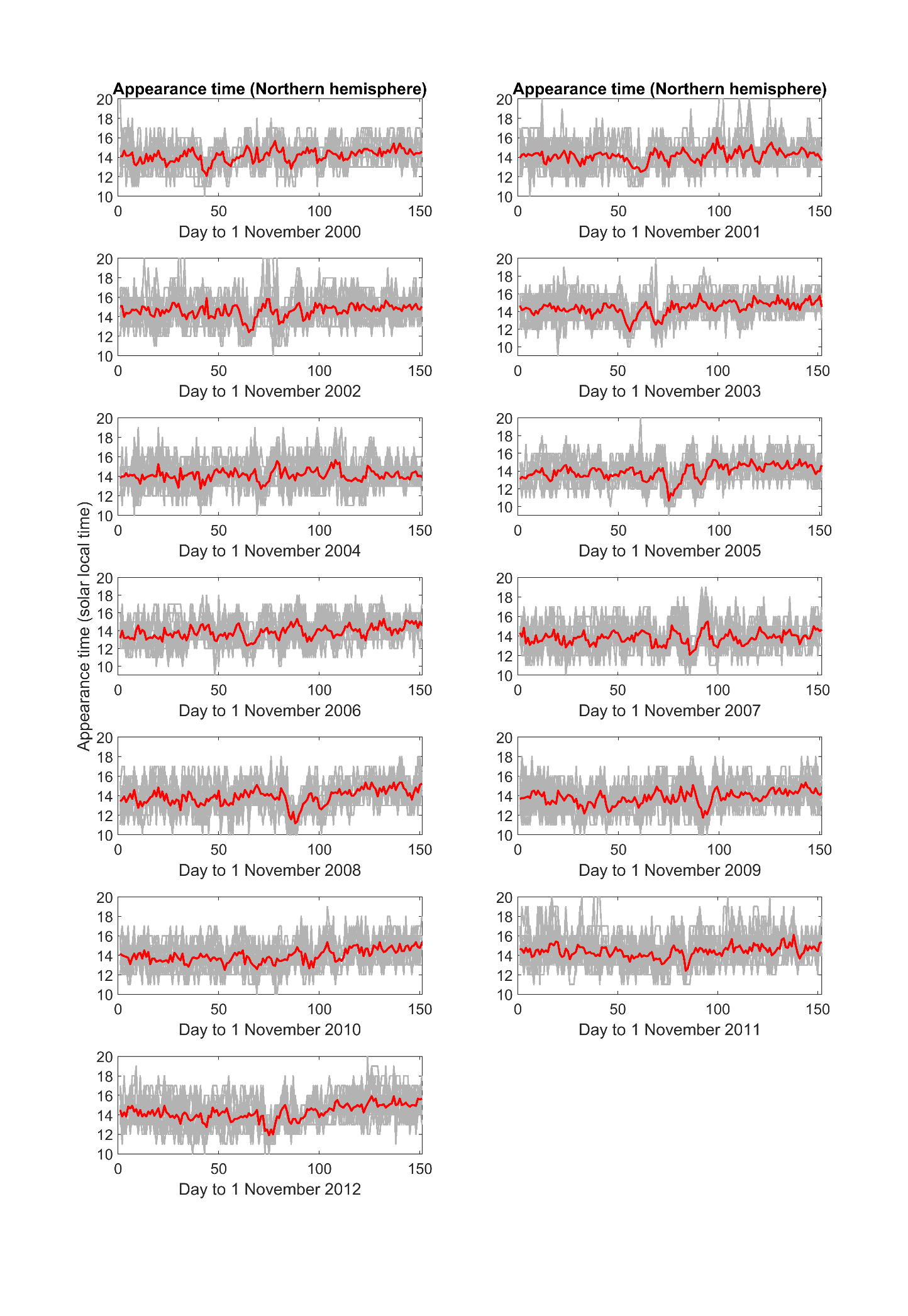


**Fig. S4** Appearance time of Northern EIA crests from November to March during 2000–2013. Gray curves denote EIA crest time of each longitude. Red curves represent the zonal mean EIA crest time.

1. **Equatorial ionization anomaly crest**

EIA crests are extracted from each LTT by determined the maximum TEC during 08:00 SLT – 20:00 SLT, and daily EIA crest time is averaged over the 24 longitudes. Figure S4 displays the whole EIA crest time during the 14-year period. Figure S5 further shows that the binned EIA crest times versus lunar phase (i.e. the solid and dotted line in Fig. 2a), which shows prominent semimonthly periodicity.

1. **Odds**

In this study, there are 12 SSWs during the 14-year period. Each statistical experiment is consisted of 24 and 312 EIA crests time in the SSW and reference year, respectively. Therefore, 4032 (=336x12) EIA crests time are included in odds estimation (Fig. 5).


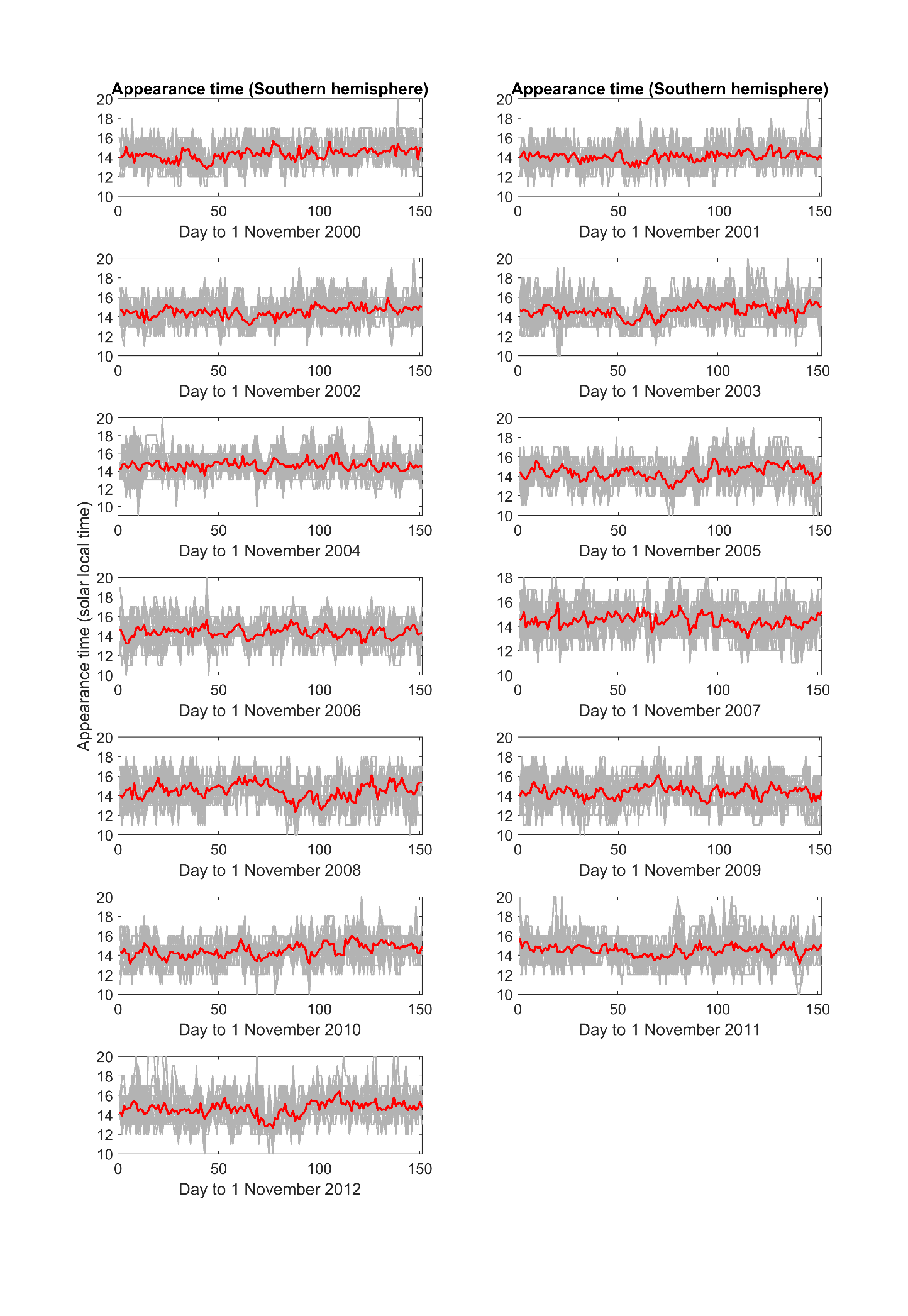


**Fig. S5** Appearance time of Southern EIA crests from November to March during 2000–2013.

1. **Appearance times of EIA crests versus various lunar phases**


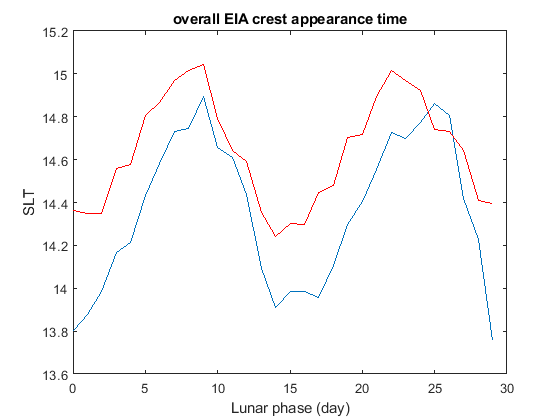


**Fig. S6**  Appearance times of EIA crests in the Northern (blue) and Southern (red) Hemispheres versus various lunar phases. The blue and red curves are extracted from Fig. 2.

1. **LTT plots of each comparison year**

To clarify the LTT plots on the same lunar phase day as the 12 CP07 SSW events (Fig. 4), Figure S7 display the ΔTEC LTT plots of each comparison year (i.e., corresponding reference; non SSW day). ΔTEC (=TEC_SSW_-TEC_ref_). TEC_ref_ is constructed by a moving median at each solar local time (SLT) 7 days before and after a certain observation day (i.e., SSW day in Figure 3 and non SSW day in Figure S7). Figure S7 illustrates detailed ΔTEC of each SSW event (Fig. 3) and its comparison year (i.e., non SSW day). In fact, Figure 4 is the average over Figure S7 for each event.


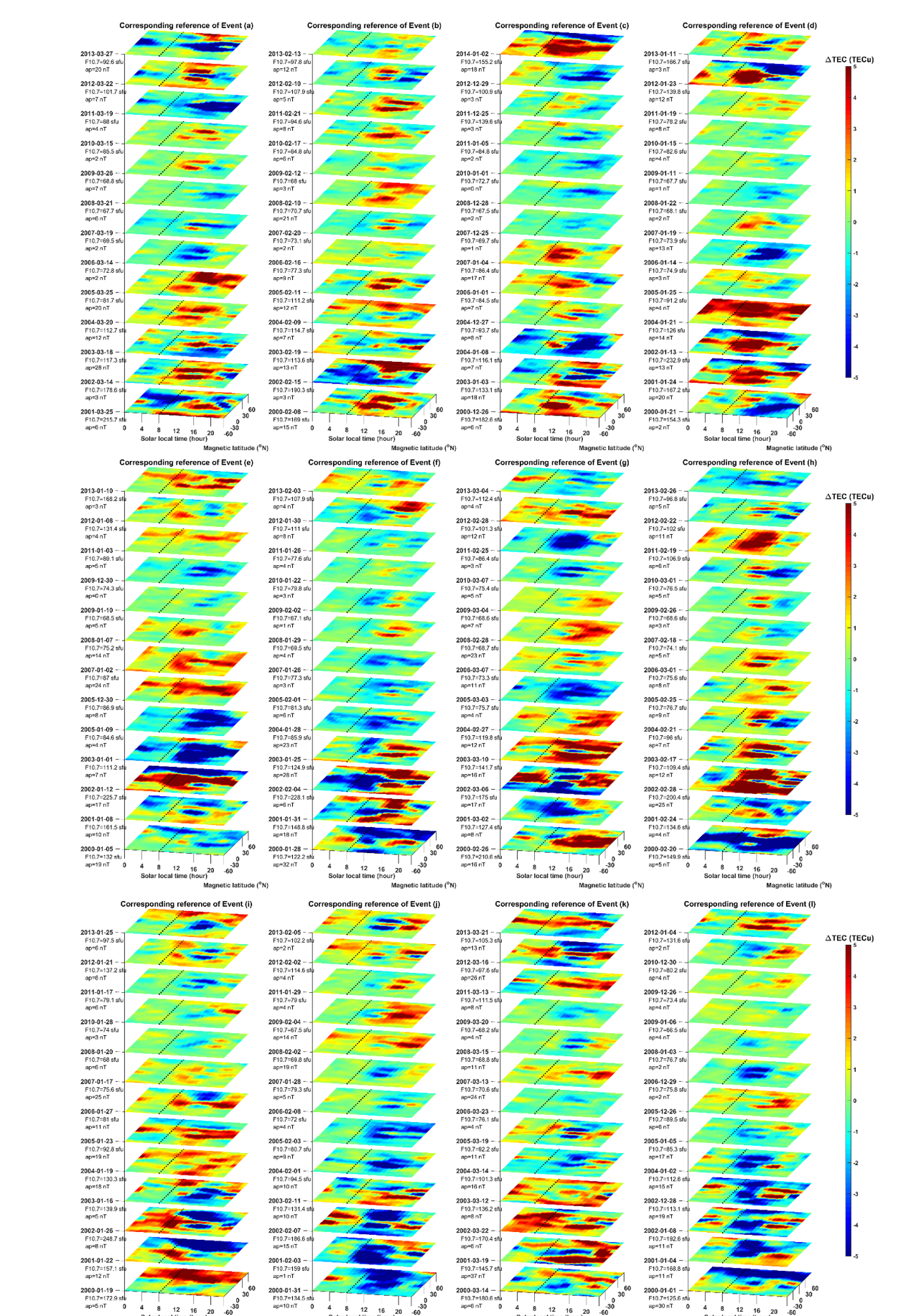


**Fig. S7** Detailed LTT plots of ΔTEC in corresponding reference years for each SSW event.

1. **Comparison of EIA crest time between with and without SSW**

Figure S8 detailedly illustrates the statistical results in Figure 5 and Table 1, which shows that the appearance time of EIA crest on the reference days (i.e., without the SSW event days) generally is a function of lunar phase (the red curve shows the lunar phase advancing EIA by about 1 hour), while the SSW events can further advance the appearance time by about 0.47 hour. Thus, SSWs can individually result in the early appearance of EIA crests, regardless of the lunar phase.

**
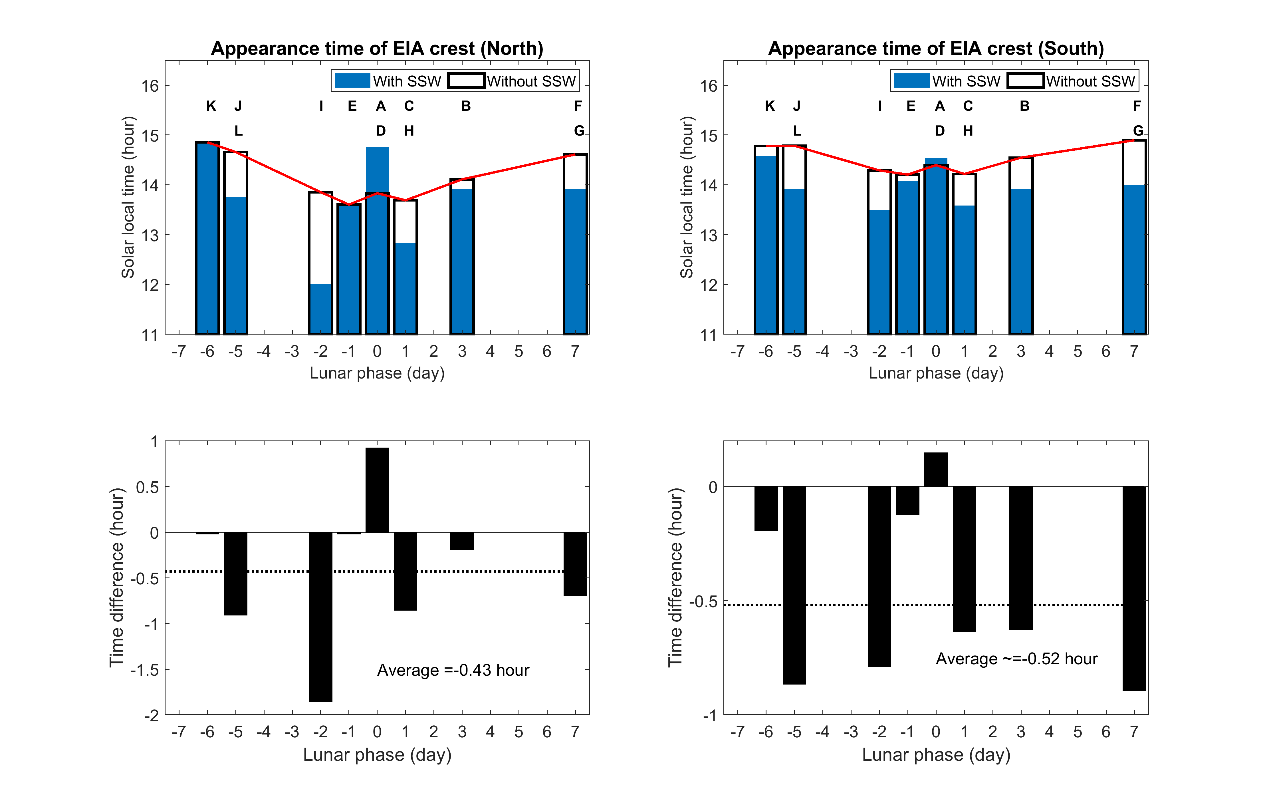
**

**Fig. S8** The bar charts of the mean of EIA crest appearance times on day with the SSW events (blue bar) and those without (i.e., the reference; open rectangular bar). The red curve denotes the top of open rectangular bars. Left (Right) panels display the mean time of EIA crests and the difference (black bar) on various lunar phase day of the events in the Northern (Southern) Hemisphere. The SSW effects can individually advance EIA appearance time by about 0.47 (=(0.43+0.52)/2) hours.

| 1. **SSW Events and Their Reference Dates**   **Table S1.** SSW Events and Their Reference Dates during 2000–2013 | | | |
| --- | --- | --- | --- |
| Event | yyyy/mm/dd | Lunar Phase (day) | Reference Date (yyyy/mm/dd) |
| **A** | **2000/03/20** | **0** | 2001/03/25  2002/03/14  2003/03/18  2004/03/20  2005/03/25  2006/03/14  2007/03/19  2008/03/21  2009/03/26  2010/03/15  2011/03/19  2012/03/22  2013/03/27 |
|  |  |  |  |
| **B** | **2001/02/11** | **3** | 2000/02/08  2002/02/15  2003/02/19  2004/02/09  2005/02/11  2006/02/16  2007/02/20  2008/02/10  2009/02/12  2010/02/17  2011/02/21  2012/02/10  2013/02/13 |
|  |  |  |  |
|  |  |  |  |
|  |  |  |  |
| **C** | **2001/12/31** | **1** | 2000/12/26  2003/01/03  2004/01/08  2004/12/27  2006/01/01  2007/01/04  2007/12/25  2008/12/28  2010/01/01  2011/01/05  2011/12/25  2012/12/29  2014/01/02 |
|  |  |  |  |
| **D** | **2003/01/18** | **0** | 2000/01/21  2001/01/24  2002/01/13  2004/01/21  2005/01/25  2006/01/14  2007/01/19  2008/01/22  2009/01/11  2010/01/15  2011/01/19  2012/01/23  2013/01/11 |
|  |  |  |  |
| **E** | **2004/01/06** | **-1** | 2000/01/05  2001/01/08  2002/01/12  2003/01/01  2005/01/09  2005/12/30  2007/01/02  2008/01/07  2009/01/10  2009/12/30  2011/01/03  2012/01/08  2013/01/10 |
|  |  |  |  |
| **F** | **2006/01/21** | **7** | 2000/01/28  2001/01/31  2002/02/04  2003/01/25  2004/01/28  2005/02/01  2007/01/26  2008/01/29  2009/02/02  2010/01/22  2011/01/26  2012/01/30  2013/02/03 |
|  |  |  |  |
| **G** | **2007/02/24** | **7** | 2000/02/26  2001/03/02  2002/03/06  2003/03/10  2004/02/27  2005/03/03  2006/03/07  2008/02/28  2009/03/04  2010/03/07  2011/02/25  2012/02/28  2013/03/04 |
|  |  |  |  |
|  |  |  |  |
|  |  |  |  |
| **H** | **2008/02/22** | **1** | 2000/02/20  2001/02/24  2002/02/28  2003/02/17  2004/02/21  2005/02/25  2006/03/01  2007/02/18  2009/02/26  2010/03/01  2011/02/19  2012/02/22  2013/02/26 |
|  |  |  |  |
| **I** | **2009/01/24** | **-2** | 2000/01/19  2001/01/22  2002/01/26  2003/01/16  2004/01/19  2005/01/23  2006/01/27  2007/01/17  2008/01/20  2010/01/28  2011/01/17  2012/01/21  2013/01/25 |
|  |  |  |  |
| **J** | **2010/02/09** | **-5** | 2000/01/31  2001/02/03  2002/02/07  2003/02/11  2004/02/01  2005/02/03  2006/02/08  2007/01/28  2008/02/02  2009/02/04  2011/01/29  2012/02/02  2013/02/05 |
|  |  |  |  |
| **K** | **2010/03/24** | **-6** | 2000/03/14  2001/03/19  2002/03/22  2003/03/12  2004/03/14  2005/03/19  2006/03/23  2007/03/13  2008/03/15  2009/03/20  2011/03/13  2012/03/16  2013/03/21 |
|  |  |  |  |
| **L** | **2013/01/06** | **-5** | 2000/01/01  2001/01/04  2002/01/08  2002/12/28  2004/01/02  2005/01/05  2005/12/26  2006/12/29  2008/01/03  2009/01/06  2009/12/26  2010/12/30  2012/01/04 |
